# Performance of Scores Predicting Adverse Outcomes in Procurement Kidney Biopsies From Deceased Donors With Organs of Lower-Than-Average Quality

**DOI:** 10.3389/ti.2023.11399

**Published:** 2023-10-12

**Authors:** Florian G. Scurt, Angela Ernst, Carl-Ludwig FischerFröhlich, Anke Schwarz, Jan U. Becker, Christos Chatzikyrkou

**Affiliations:** ^1^ Faculty of Medicine, University Hospital Magdeburg, Magdeburg, Germany; ^2^ University Hospital of Cologne, Cologne, Germany; ^3^ Deutsche Stiftung Organtransplantation, Frankfurt am Main, Germany; ^4^ Hannover Medical School, Hanover, Germany; ^5^ PHV Dialysis Center, Halberstadt, Germany

**Keywords:** kidney transplantation, pathology, transplant loss, marginal donor, procurement biopsies

## Abstract

Several scores have been devised for providing a prognosis of outcomes after kidney transplantation. This study is a comprehensive test of these scores in a cohort of deceased donors with kidneys of lower-than-average quality and procurement biopsies. In total, 15 scores were tested on a retrospective cohort consisting of 221 donors, 223 procurement biopsies, and 223 recipient records for performance on delayed graft function, graft function, or death-censored graft loss. The best-performing score for DGF was the purely clinical Chapal score (AUC 0.709), followed by the Irish score (AUC 0.684); for graft function, the Nyberg score; and for transplant loss, the Snoeijs score (AUC 0.630) and the Leuven scores (AUCs 0.637 and 0.620). The only score with an acceptable performance was the Chapal score. Its disadvantage is that knowledge of the cold ischemia time is required, which is not known at allocation. None of the other scores performed acceptably. The scores fared better in discarded kidneys than in transplanted kidneys. Our study shows an unmet need for practical prognostic scores useful at the time of a decision about discarding or accepting deceased donor kidneys of lower-than-average quality in the Eurotransplant consortium.

## Graphical Abstract

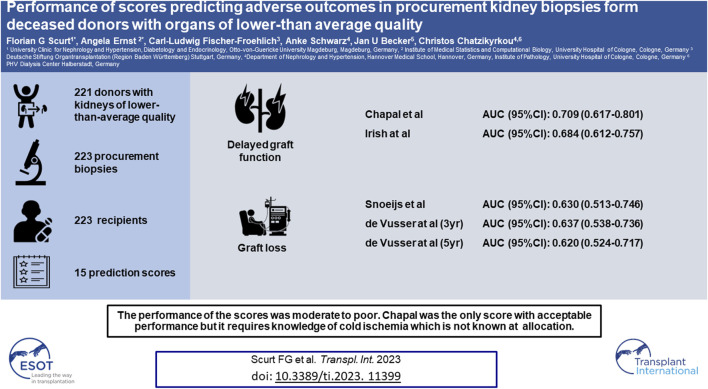

## Introduction

For most patients with end-stage kidney disease, kidney transplantation is the best available treatment with better survival, quality of life and lower use of healthcare resources [1–3]. Despite the increasing use of living donation [4, 5], most patients on dialysis still have to wait on a deceased donor kidney transplant (DDK). Today, transplant physicians are facing the dilemma of how to best use the scarce pool of increasingly older DDKs while avoiding the risk of a poor outcome for the recipients which can be associated with delayed graft function (DGF), premature transplant loss or even endanger their lives [1, 3].

Several purely clinical [6–13], combined clinicohistological [14–16], or purely histological scores [17–20] have been devised for quality assessment of DDKs; the Nyberg score, is for practical purposes best considered clinical, as it does not requires histopathology [9]. The scores with a histology component have been developed on preimplantation but not the clinically decisive procurement biopsies from unselected cohorts, reflecting the full spectrum of DDK quality, including those with the lowest risk. Some of these scores have been internally [9, 14, 16] or externally validated in the publications of subsequent scores from other authors or in separate studies. A recent publication has tested four scores [6–8, 12] for their performance in the prognostication of DGF in a large Dutch cohort of unselected preimplantation biopsies [21]. An earlier study from the United Kingdom evaluated the performance of four scores [9, 11, 22, 23] regarding mid-term transplant function [24], two of which have been updated since [7, 9]. A recent study from the United States (US) validated three scores [9, 25, 26] on a single-centre cohort of donors with kidneys of lower quality for the prognostic performance regarding two-year-transplant survival [27]. Similarly, in another study [28], four scores, including that proposed by Banff [16, 19, 25, 29] failed to predict graft survival and early graft function. The scores and their validation studies have helped to better understand and address the causes of DGF and premature transplant failure. However, these scores have never been validated regarding their usefulness for the decision about acceptance or discard of a DDK on a set of procurement biopsies, taken to assess organ quality before allocation. This is particularly important in view of recent data showing that procurement biopsies lead to discard of organs suitable of transplantation [30].

Primary aim of this study is to conduct the overdue comprehensive test of a variety of scores (listed in Table 1) for their performance on various end points, such as delayed graft function, graft function, or death-censored graft loss on a retrospective cohort of procurement biopsies specifically commissioned for DDK quality assessment by the Deutsche Stiftung Organtransplantation (DSO; German Foundation for Organ Transplantation), operating within the Eurotransplant consortium. As a secondary aim, we examined whether purely clinical scores perform as well as scores including a histopathology component. Lastly, we wanted to test their performance on the considerable proportion of the discarded kidneys in our cohort.

**TABLE 1:** Parameters used in the previously published scores for the quality assessment of DDKs.

Score		Donor								Transplant procedure						Donor kidney									Recipient						
Name	Type	Cause of death	Hypertension	DM	Kidney function	Age	Ethnicity	Weight	HCV	CIT	WIT	DKT	MM	CMV match	Induction therapy with ATG	Global GS	Banff ci	Banff ct	Banff cv	Banff cg	Banff i	Banff ah	Banff mm	Renal artery plaque	Age	Weight	Body mass index	Previous Tx	DM	Dialysis duration	PRA
*Balaz* ^15^	C + P	x				x											x		x												
Chapal^6^	C				x	x				x					x												x				
Irish^7^	C				x	x		x		x	x		x													x				x	x
Jeldres^8^	C					x				x			x												x	x					x
Schold^11^	C	x	x	x		x	x			x			x	x																	
*Navarro* ^17^	P															x	x	x	x			x									
Port^10^	C	x	x		x	x																									
Rao^26^	C	x	x	x	x	x	x		x	x		x	x																		
*Snoeijs* ^20^	P															x	x	x	x	x		x									
de Vusser^16^	C + P					x											x	x													
Remuzzi^19^	P															x	x	x	x												
*Anglicheau* ^14^	C + P		x		x											x															
Nyberg^9^	C	x	x	x	x					x														x							
*Ortiz* ^18^	P															x	x	x	x		x	x	x								
Foucher^13^	C																								x			x	x	x	

Abbreviations: ah, arteriolar hyalinosis; ATG, anti-thymocyte globulin; BMI, body mass index; CIT, cold ischemia time; ci, interstitial fibrosis; cg, glomerular basement membrane splitting; CMV, cytomegalovirus; ct, tubular atrophy; cv, arterial intimal fibrosis; DDK, deceased donor kidney; DKT, double kidney transplantation; DM, diabetes mellitus; EPTS, Estimated post transplant survival score; HCV, hepatitis C virus; GS, glomerulosclerosis; i, interstitial infiltrates; KDRI, kidney donor risk index; MM, miss matches, mm mesangial matrix; PRA, panel-reactive antibodies; WIT, warm ischemia time.

The score designation and the reference are given in the first column; the type of score as in purely clinical (C), combined clinical and pathological (C + P) or solely pathological (P) is given in the second column. Subsequent columns list the parameters used in the respective scores. The parameters are organized as relating to the donor, to the transplant procedure, to the transplant itself or to the recipient. Note that although renal artery plaque as used in the Nyberg score is a pathological finding, it is not typically assessed by a pathologist (pathological and clinic-pathological scores are in italics; the numbers correspond to the references in the manuscript).

## Materials and Methods

### Biopsies, Reporting, Donor, and Recipient Data

We extracted data from the “DSO Region Nord” and from the German transplant centers of kidneys allocated, between 1 January 2003, and 31 March 2012. The collection of recipient follow-up data was completed in December 2015. Data were analyzed between 1 January 2018, and 31 May 2020. Only adult recipients of deceased donor kidneys of lower quality were included. Recipients with dual kidney- and combined kidney transplantation were excluded. Our cohort consisted exclusively of brain death donors since donation after cardiac death is not allowed in Germany.

The allocation was under the auspices of Eurotransplant, an international non-profit organization responsible for the coordination and distribution of organs for transplantation between residents of eight European countries.^1^

The following donor data were collected: age, sex, weight, height, body mass index (BMI), length of hospital stay, cardiopulmonary resuscitation, cardiovascular comorbidities, history of smoking, cause of brain death, use of vasopressors, hemodynamic parameters such as, blood pressure and central venous pressure, creatinine at admission, peak creatinine and creatinine at organ recovery, diuresis volume 24 h and at the last hour before recovery, and urine dipstick test at recovery. Recipients’ records were searched for medical history, immunologic risk, peritransplant data, and outcome.

**FIGURE 1:**
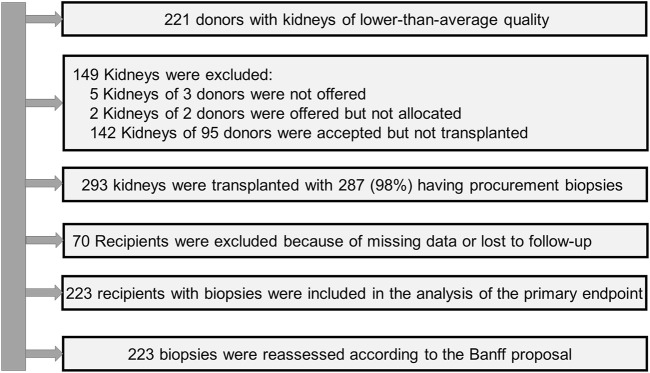
Flow chart of the study (DGF, delayed graft function; m, months; PNF, primary non-function; yr, year).

The biopsies were evaluated at the Institute of Pathology in parallel to the transport of the DDK and the preparation for transplantation. Procurement biopsies were not performed in all kidneys but only in that deemed to be of lower quality to increase their chance of acceptance. The results were reported after rapid paraffin-embedding on multiple hematoxylin-eosin and periodic-acid-Schiff-stained sections within 4 h. The DSO oversaw DDK management after notification. The decision about use or discard of the DDK was then made by the transplant physician in the receiving centre. The first assessment was done by the pathologist on duty and included information on representativeness of biopsy, number of glomeruli and arteries, percentage of tubular atrophy, and grading of acute tubular injury. The recommendation was usually suitable/not suitable or partially suitable. The histopathological scores reported below were provided in a second, blinded reading by an experienced nephropathologist. A flowchart of the study is given in Figure 1.

Histopathological parameters included type of biopsy (needle or wedge), total number of glomeruli, ratio of globally sclerosed glomeruli, number of arteries (media ≥2 smooth muscle cell layers), presence of focal and segmental glomerulosclerosis (FSGS), Banff Lesion Scores i, t, v, g, ptc, ci, ct, cv, cg, ah, arteriolar fibrosis scored as absent, mild, moderate and severe, cortical tubular hypertrophy, epithelial cell flattening, brush border loss, vacuolisation and luminal detritus scored as 0 (absent), 1 (<25%), 2 (<50%) and 3 (≥50%), tubular nuclear loss scored as 0 (absent), 1 (1 quadrant), 2 (two quadrants), 3 (3 quadrants of the most affected tubular cross-section), pyelonephritis and thrombotic microangiopathy. The Banff meeting report 2011, the Banff consensus criteria for preimplantation biopsies, the german recommendations for procurement biopsies [29, 31, 32] and classification systems for glomerular diseases [33, 34], as well as scoring systems for calcification [35] and acute tubular injury [36, 37] were also considered. A summary of all histopathological parameters is provided in Supplementary Material.

### Definitions

The definition of lower organ quality depended not on strict criteria but was based on clinical judgment considering the macroscopic appearance of the organ in combination with donor’s clinical data. The macroscopic appraisal was done on the “back table,” after removal of the perinephric fat and the clean dissection of the vessels from the surrounding tissues. It included organ quality as well as perfusion quality, both of which were rated as good, medium, or poor; likewise, atherosclerosis was characterized as no, mild, or severe. The decision was usually felt after discussion of each case between the senior surgeon of the harvesting team and the physician of the recipient’s center. Senior surgeons were accredited by the DSO and had many years of experience in the transplant field.

Extended criteria donors (ECD) were classified as previously reported [38]. eGFR was calculated by means of the Chronic Kidney Disease Epidemiology Collaboration (CKD-EPI) equation. Admission, highest, lowest, and terminal eGFR were respectively estimated by using the first, the lowest, the highest and the last serum creatinine prior to organ recovery [39]. Primary non-function (PNF) was defined as the permanent lack of graft function from the time of transplantation [40] and delayed graft function (DGF) as the need for dialysis in the first week [41].

### Scores

An overview of the parameters included in the respective scores is given in Table 1. Kidney Donor Profile and Risk Index (KDP, KDRI) were calculated according to the Organ Procurement and Transplantation Network (OPTN)^2^ and estimated post transplant survival (EPTS) score by the web calculator provided by OPTN (EPTS calculator—OPTN).^3^

### Outcome Measures

The following outcomes were analyzed: PNF, DGF, graft function at 3 months, one- and 3 years, death censored graft failure and patient death at one, three and 5 years. All survival times were censored at the last date a patient was known to be alive. eGFR results were presented as 10 mL/min per 1.73 m^2^ for ease of interpretation.

### Statistics

Continuous variables were described as mean ± standard deviation (SD) and central trends between groups compared by Mann-Whitney-U-tests. Fisher’s exact- and χ^2^-tests were used to compare distributions of categorical variables, respectively. To estimate how well a risk-score discriminates the different endpoints, the area underneath the receiver operating characteristics curve (AUC) was calculated. AUCs range from 0% to 100%, with 0% suggesting perfect inaccuracy, 100% perfect accuracy, 50% suggesting no discrimination and 50%–70% suggesting poor discrimination, 70%–80% suggesting acceptable and 80%–90% excellent and finally 90% suggesting outstanding performance [42, 43]. A *p*-value below 0.05 was considered significant in all comparisons in two-sided tests; however, in this retrospective observational study, *p*-values can only be considered descriptive. Statistical analysis was performed with the use of SPSS software, v24 (IBM Corp, Armonk, NY, United States) and IBM SPSS Statistics Essentials for R.

### Ethical Permission

All organ transplants were performed according the Declaration of Istanbul [44]; no transplants from prisoners were used. The study was conducted in accordance with the Declarations of Helsinki and approved by the local ethical review board of Hannover Medical School (No. 1519-2012).

## Results

### Donors’ and Recipients’ Characteristics

From 442 kidneys recovered from 221 donors, 149 were discarded. In 287 (98%) of the 293 transplanted kidneys the tissue blocks were found. Follow-up data were available from 223 recipients (Figure 1). The KDRI was 1.48 and 107 (63.3%) were ECD. The average age was 61 years and 54% were males. Only 13% of donors had diabetes and 30% cardiovascular disease. The prevalence of hepatitis B and C was low (6.5% and 1.2%). Cerebrovascular accident was the most common cause of brain death (60%). The serum creatinine at recovery was 149 μmol/L. Approximately 50% of donors experienced acute kidney injury (AKI) (Table 2). The accepted kidneys showed macroscopically a good perfusion and organ quality at all, except for atherosclerosis which was severe in 46.5% of them. Biopsies were performed in 80% and the majority were needle biopsies with a representative number of glomeruli and arteries. Mean and minimal (<5%) global glomerulosclerosis were 10.4% and 50% respectively, whereas the majority of acute and chronic tubular, interstitial, and vascular Banff lesion scores were of low grade. On the contrary, acute tubular injury was, as expected, more severe (Table 3). The average age of recipients was 61 years. They showed a low immunologic risk profile, a cold ischemia time (13.8 h) which was at the lower range of that reported for Eurotransplant [45] and a high EPTS score (Table 4). PNF occurred in 26 (11.7%) and DGF in 109 (48.9%) patients. We observed 49 graft losses during a median follow-up of 43.8 months (IQR 19–68 months). Patient and death-censored graft survival at 1, 3, and 5 years after kidney transplantation were respectively 90.6% and 91.1%, and 86.1% and 82.9% and 83% and 81.6% (Table 5).

**TABLE 2:** Demographic data and ICU monitoring parameters of the donors.

Characteristic	Value
Donor characteristics (No. of donors = 169)	
Age, y	60.8 ± 16.2
Sex, n (%)	
Female	77 (45.6)
Male	92 (54.4)
BMI, kg/m^2^	27.4 ± 5.8
Diabetes mellitus, n (%)	22 (13.0)
Hypertension, n (%)	96 (56.8)
Cardiovascular disease, n (%)	49 (29.2)
Smoker, n (%)	46 (27.2)
Hepatitis B Virus positive, n (%)	11 (6.5)
Hepatitis C Virus positive, n (%)	2 (1.2)
Cytomegalovirus positive, n (%)	110 (65.1)
Cerebrovascular accident (CVA), n (%)	101 (59.8)
Extended Criteria Donors (ECD), n (%)	107 (63.3%)
Kidney Donor Risk Index (KDRI)	1.48 ± 0.51
KDRI Grading, n (%)	
KDRI Grade I (0–20)	11 (6.5)
KDRI Grade II (21–40)	17 (10.1%)
KDRI Grade III (41–60)	29 (17.2)
KDRI Grade IV (61–80)	27 (16.0)
KDRI Grade V (81–100)	85 (50.3)
Donor ICU data	
Time ICU until confirmed brain death, h	118.1 ± 126.6
Time confirmed brain death until cross-clamp, h	13.2 ± 14.8
CPR at ICU stay, n (%)	30 (17.8)
Transfusion at ICU stay, n (%)	18 (10.7)
Units of RBC, n (%)	3.06 ± 8.13
Units of FFP, n (%)	3.28 ± 9.28
Volume expander at ICU stay, n (%)	25 (14.8)
Diuretics at ICU stay, n (%)	22 (13.1)
Antidiuretics at ICU stay, n (%)	57 (33.9)
Antibiotics at ICU stay, n (%)	90 (53.3)
AKI, n (%)	79 (46.7)
RIFLE criteria, n (%)	
No AKI	90 (53.3)
Risk	46 (27.2)
Injury	13 (7.7)
Failure	20 (11.8)
Serum creatinine, µmol/L	
At admission	111 ± 88
Minimum	101 ± 73
Peak	161 ± 129
Last	149 ± 119
Last blood and urine values before cross clamp	
Hemoglobin, g/dL	17.5 ± 3.4
White cell count, per cubic millimeter	13.58 ± 5.052
Platelet count, per cubic millimeter	176,868 ± 97,578
International normalized ratio	1.27 ± 0.51
Activated partial thromboplastin time, sec	39.4 ± 16.6
Aspartate transaminase, IU/L	123.4 ± 293.4
Alanine transaminase, IU/L	109.2 ± 357.4
Alkaline phosphatase, IU/L	98.3 ± 51.2
Lactate dehydrogenase, IU/L	456.9 ± 500.6
Total bilirubin, mg/dL	16.7 ± 16.6
C-reactive protein, mg/L	187.8 ± 191.6
Urine protein dipstick, % (neg/1+/2+)	64.1/29.9/6.0
Urine volume last 24 h, mL/kg	42.3 ± 32.4
Urine volume last hour, mL/kg	2.44 ± 5.93
Donor data at cross-clamp period	
Time incision until cross-clamp, min	52.6 ± 31.0
Time cross-clamp until ectomy right	43.7 ± 16.6
Time cross-clamp until ectomy left	49.0 ± 17.9
Catecholamines, n (%)	130 (76.9)
Mean Arterial Blood pressure, mmHg	97.2 ± 15.8
Pulse,/min	96.4 ± 25.6
Central venous pressure, mmHg	9.74 ± 3.69
Temperature, °C	36.61 ± 1.17

Continuous parameters are given as mean ± standard deviation, numerical and ordinal parameters as count and percentage.

Abbreviations: AKI, acute kidney injury; BMI, body mass index; DDK, deceased donor kidney; DGF, delayed graft function; FFP, fresh frozen plasma; HBsAG, hepatitis B virus surface antigen; ICU, intensive care unit; IU, international units; RBC, red blood cells.

**TABLE 3:** Macroscopic and histopathological parameters.

Characteristics	Transplanted kidneys (*n* = 223)
Macroscopic parameters^a^	
Perfusions quality, % (good/medium/bad)	94.6/3.1/2.2
Organ quality, % (good/medium/bad)	74.0/24.2/1.8
Atherosclerosis, % (no/mild/severe)	38.4/15.2/46.5
Organ localization, % (right kidney/left kidney)	48.4/51.6
Histopathological parameters	
Biopsy performed, n (%)	179 (80.3)
Art of biopsy, % (Needle/Wedge)	82.1/17.9
Renal cortex proportion of total parenchyma, %	66.1 ± 34.2
Glomeruli, *n*	36.2 ± 69.0
Arteries, *n*	8.1 ± 15.2
Global glomerulosclerosis, % of total glomeruli	10.4 ± 15.0
Global glomerulosclerosis < 5, %	50.3
Any FSGS, % of biopsies	2.5
Banff Lesion Scores (0/1/2/3), %	
Interstitial inflammation (i)	84.2/14.6/1.2/0.0
Tubulitis (t)	88.9/11.1/0.0/0.0
Intimal arteritis (v)	99.4/0.6/0.0/0.0
Glomerulitis (g)	86.0/12.3/1.1/0.6
Peritubular capillaritis (ptc)	100.0/0.0/0.0/0.0
Interstitial fibrosis (ci)	80.1/18.1/1.2/0.6
Tubular atrophy (ct)	61.4/36.8/1.2/0.6
Vascular fibrous Intimal thickening (cv)	41.5/33.9/21.1/3.5
Glomerular basement membrane splitting (cg)	97.1/2.9/0.0/0.0
Mesangial matrix expansion (mm)	81.9/14.0/1.8/2.3
Arteriolar hyalinosis (ah)	35.1/38.6/22.8/3.5
Interstitial fibrosis and tubular atrophy, % (0–10/10–25/25–50/>50)	70.4/14.3/13.9/0.9
Arteriolar wall fibrosis, % (no/mild/moderate/severe)	54.4/35.1/9.4/1.2
RPS diabetic nephropathy class ≥1, %	5.2
Thrombotic microangiopathy, %	6.4
Nephrocalcinosis, % (no/mild/moderate to severe)	88.9/4.7/6.4
Tubular hypertrophy, %	19.3
Epithelial cell flattening (0/1/2/3), %	3.5/40.4/32.7/23.4
Brush border membrane defect (0/1/2/3), %	1.2/26.9/46.8/25.1
Vacuolization (0/1/2/3), %	7.0/22.8/22.2/48.0
Loss of nuclear staining (0/1/2/3), %	1.8/27.5/38.0/32.7
Cellular detritus (0/1/2/3), %	15.8/40.9/23.4/19.9
Pyelonephritis, %	8.2

Continuous parameters are given as mean ± standard deviation, numerical and ordinal parameters as count and percentage.

Abbreviations: DDK, deceased donor kidney; FSGS, focal and segmental glomerulosclerosis; RPS, renal pathology society.

^a^ Of note, macroscopic parameters listed in this table were determined by the harvesting surgeon, and not by a pathologist while the histopathological parameters were determined retrospectively by an experienced nephropathologist.

**TABLE 4:** Clinical parameters of recipients.

	Recipients with follow-up data (*n* = 223)
Recipients’ parameters	
Age, y	61.0 ± 13.5
Sex, n (%)	
Female	75 (33.6)
Male	148 (66.4)
BMI, kg/m^2^	25.5 ± 4.4
Diabetes mellitus, n (%)	59 (26.5)
Hypertension, n (%)	191 (85.7)
Cardiovascular disease, n (%)	96 (43.0)
HBsAg positive, n (%)	49 (22.0)
Hepatitis C Virus positive, n (%)	6 (2.7)
Cytomegalovirus positive, n (%)	148 (66.4)
Dialysis vintage, months	166.9 ± 79.2
Prior organ transplant, n (%)	24 (10.8)
Estimated Post Transplant Survival (EPTS)	2.66 ± 0.62
Estimated Post Transplant Survival (EPTS) Groups, n (%)	
Group 1: 0%–20%	18 (8.1)
Group 2: 21%–40%	14 (6.3)
Group 3: 41%–60%	29 (13.0)
Group 4: 61%–80%	30 (13.5)
Group 5: 81%–100%	131 (58.7)
Transplant baseline parameters	
HLA-A mismatch (0/1/2), %	14.3/56.1/29.6
HLA-B mismatch (0/1/2), %	8.1/48.9/43.0
HLA-DR mismatch (0/1/2), %	14.3/55.2/30.5
Negative PRA at transplantation, n (%)	200 (89.7)
Average PRA at transplantation, %	2.4 ± 9.7
Historic Peak of PRA, %	7.5 ± 20.8
Origin of donor kidney (right/left/both), %	50.7/48.0/1.3
Cold ischemia time, h	13.8 ± 5.0
Warm ischemia time, min	40.6 ± 14.3
Maintenance therapy	
Calcineurin inhibitors, % (Cyclosporin/Tacrolimus/other)	74.8/24.5/0.7
Anti-metabolites, % (Azathioprine/Mycophenolate/other)	0.7/84.1/15.2
mTOR inhibitors, %	4.2
Steroids, %	91.0

Continuous parameters are given as mean ± standard deviation, numerical and ordinal parameters as count and percentage.

Abbreviations: b, both; BMI, body mass index; HBsAg, hepatitis b virus surface antigen; HLA, human leukocyte antigen; PRA, panel reactive antibodies.

**TABLE 5:** Outcome data of recipients.

	Recipients with follow-up data (*n* = 223)
Primary non function, %	26 (11.7)
Delayed graft function, %	109 (48.9)
Patient survival at 1 year, %	202 (90.6)
Patient survival at 3 years, %	192 (86.1)
Patient survival at 5 years, %	185 (83.0)
Death-censored graft survival at 1 year, %	163 (91.1)
Death-censored graft survival at 3 years, %	141 (82.9) (n_missing_ = 6)
Death-censored graft survival at 5 years, %	133 (81.6) (n_missing_ = 6)
Kidney function at 3 months (creatinine), µmol/L	188.3 ± 77.9 (n_missing_ = 1)
Kidney function at 3 months (eGFR), mL/min/1.73 m^2^	34.6 ± 14.7 (n_missing_ = 1)
Kidney function at 1 year (creatinine), µmol/L	166.9 ± 52.9 (n_missing_ = 1)
Kidney function at 1 year (eGFR), mL/min/1.73 m^2^	37.4 ± 13.6 (n_missing_ = 1)
Kidney function at 3 years (creatinine), µmol/L	165.8 ± 59.8 (n_missing_ = 61)
Kidney function at 3 years (eGFR), mL/min/1.73m^2^	38.4 ± 15.2 (n_missing_ = 61)
Rejections overall	0.65 ± 1.05 (n_missing_ = 93)
Without Rejections (%)	57.7

Continuous parameters are given as mean ± standard deviation, numerical and ordinal parameters as count and percentage.

### Donor and Organ Related Differences Between Discards and Transplantations

**FIGURE 2:**
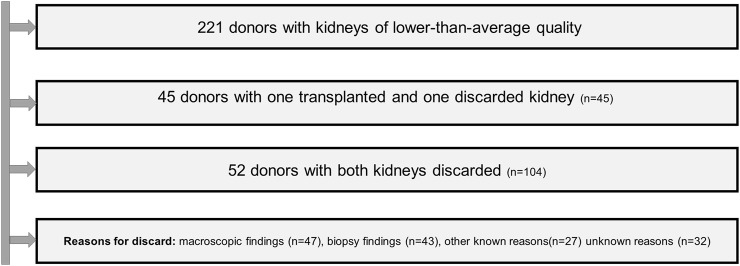
Flow chard of the handling of discarded organs.

149 of the 442 available kidneys were discarded (33%). 45 were recovered from donors whose contralateral kidney was transplanted and 104 from donors whose both kidneys were discarded (Figure 2).

Except for the higher prevalence of hepatitis C and the longer duration of brain death, there were no differences in the baseline characteristics between donors of transplanted and discarded kidneys (Table 6).

**TABLE 6:** Comparison of baseline characteristics between donors with transplanted and discarded kidneys.

	Transplanted kidneys (*n* = 293)	Discarded kidneys (*n* = 149)	*p*-value
Donor characteristics			
No of Donors	169	97	
Age, y	60.8 ± 16.2	61.4 ± 15.2	0.999
Sex, n (%)			
Female	77 (45.6)	38 (39.6)	0.345
Male	92 (54.4)	58 (60.4)	
BMI, kg/m^2^	27.4 ± 5.8	27.3 ± 5.4	0.874
Diabetes mellitus, n (%)	22 (13.0)	14 (14.6)	0.721
Hypertension, n (%)	96 (56.8)	56 (58.3)	0.809
Cardiovascular disease, n (%)	49 (29.2)	30 (31.6)	0.682
Smoker, n (%)	46 (27.2)	27 (28.1)	0.874
Hepatitis B Virus positive, n (%)	11 (6.5)	7 (7.3)	0.808
Hepatitis C Virus positive, n (%)	2 (1.2)	6 (6.3)	**0.021**
Cytomegalovirus positive, n (%)	110 (65.1)	65 (67.5)	0.665
Cerebrovascular accident (CVA), n (%)	101 (59.8)	50 (52.1)	0.225
Kidney Donor Risk Index (KDRI)	1.48	1.52	0.788
Time confirmed brain death until cross-clamp, h	13.2 ± 14.8	15.8 ± 17.1	**0.032**
AKI, n (%), Creatinine first, max	61 (36.1)	32 (33.3)	0.651
AKI, n (%), Creatinine min, max	79 (46.7)	40 (41.7)	0.424
Last serum creatinine, mg/dL	1.68 ± 1.35	1.80 ± 1.40	0.775
Last creatinine kinase	849 ± 1,046	1,608 ± 8,345	0.435
Last Sodium, mmol/L	148.0 ± 9.4	147.6 ± 7.9	0.847
Blood pressure, mmHg			
Systolic	126.1 ± 22.1	126.1 ± 25.0	0.918
Diastolic	68.0 ± 12.3	68.3 ± 14.9	0.870
Mean arterial	97.2 ± 15.8	97.3 ± 18.3	0.932
Pulse/min	96.4 ± 25.6	97.2 ± 24.1	0.889
Central venous pressure, mmHg	9.74 ± 3.69	9.5 ± 3.7	0.803
Central venous pressure—PEEP, mmHg	4.94 ± 4.60	4.7 ± 4.5	0.568
Temperature, °C	36.61 ± 1.17	36.7 ± 1.2	0.631
P_a_O_2_/FiO_2_ Ratio	252.1 ± 108.8	266.6 ± 108.7	0.425
Last urine test strip, % (neg/+/++)			
Protein	64.1/29.9/6.0	63.8/33.0/3.2	0.570
Leukocytes	56.8/27.7/15.5	53.6/34.5/11.9	0.491
Red blood cells	36.8/40.1/23.0	29.4/40.0/30.6	0.351
Nitrite	81.3/18.7/0.0	85.1/14.9/0.0	0.458
Urine volume last 24 h, mL	3,347 ± 2,272	3,372 ± 2,053	0.657
Urine volume last 24 h, mL/kg	42.3 ± 32.4	43.075 ± 29.4	0.533
Urine volume last hour, mL	194 ± 499	196 ± 288.6	0.236
Urine volume last hour, mL/kg	2.44 ± 5.93	2.53 ± 3.87	0.247

Continuous variables are presented as mean ± standard deviation.

Abbreviations: AKI, acute kidney injury; BMI, body mass index; CPR, cardiopulmonary resuscitation; DGF: delayed graft function; dl, deciliter; g, gram; h, hours; IU, international units; kg, kilogram; L, liter; mL, milliliter; min, minutes; mmHg, Millimeter of mercury; mmol, millimole; m^2^, square meter; sec; seconds; y, years; µg, microgram.

Bold values represent statistically significant parameters.

^a^ One kidney from one donor with missing data about transplantation status.

The discarded kidneys were of lower macroscopic organ quality (deemed to be bad in 9.2% vs. 1.7%, *p* < 0.001) and showed more chronic glomerular (FSGS: 9.4% vs. 2.1%, *p* = 0.013; cg3: 2% vs. 0%, *p* = 0.03), more severe acute tubular cell (cellular detritus score 3: 33% vs. 21%, *p* = 0.036), more chronic tubulointerstitial (ci, ct, IFTA *p* < 0.001) and more chronic macrovascular injury (cv ≥ 1: 75% vs. 62%, *p* = 0.02). There were no differences in the percentage of glomerulosclerosis at all (11% vs. 10%, *p* = 0.305) or other tubular cell injury features. Lastly, findings of thrombotic microangiopathy (TMA) were more often observed (14.9% vs. 5.6%, *p* = 0.001) (Table 7).

**TABLE 7:** Comparison of macroscopic and histological characteristics between transplanted and discarded kidneys.

	Transplanted kidneys (*n* = 293)	Discarded kidneys (*n* = 149)	*p*-value
Macroscopic characteristics			
Perfusions quality, (good/medium/bad), %	93.5/4.4/2.0	92.2/7.1/0.7	0.310
Organ quality (good/medium/bad) %	73.4/24.9/1.7	61.0/29.8/9.2	**<0.001**
Atherosclerosis (No/Mild/Severe), %	38.2/16.8/45.0	36.4/14.5/49.1	0.864
Histopathological characteristics			
Glomerulosclerosis, %	11.3 ± 17.2	10.1 ± 12.8	0.305
FSGS, %	2.1	9.4	**0.013**
Banff Lesion Scores (0/1/2/3), %			
Interstitial inflammation (i)	82.3/15.7/2.0/0.0	86.1/9.9/2.0/2.0	0.130
Tubulitis (t)	88.9/11.1/0.0/0.0	85.1/13.9/0.0/1.0	0.288
Intimal arteritis (v)	99.0/1.0/0.0/0.0	96.0/4.0/0.0/0.0	0.085
Glomerulitis (g)	86.4/12.1/1.0/0.5	88.1/7.9/1.0/3.0	0.244
Peritubular capillaritis (ptc)	100.0/0.0/0.0/0.0	100.0/0.0/0.0/0.0	>0.999
Interstitial fibrosis (ci)	78.8/19.2/1.5/0.5	61.4/25.7/7.9/5.0	**<0.001**
Tubular atrophy (ct)	60.6/37.4/1.5/0.5	33.7/53.5/7.9/5.0	**<0.001**
Interstitial fibrosis and tubular atrophy (IFTA), % (0–10/10–25/25–50/>50)	73.4/12.3/14.0/0.3	54.7/18.9/23.0/3.4	**<0.001**
Interstitial fibrosis and tubular atrophy (IFTA), % MW (±SD)	3.10 ± 6.80	7.39 ± 13.09	**<0.001**
Vascular fibrous Intimal thickening (cv)	38.4/37.9/20.2/3.5	24.8/43.6/26.7/5.0	0.120
cv ≥ 1	61.6	75.2	**0.018**
GBM double contours (cg) (0/1/2/3)	97.0/3.0/0.0/0.0	98.0/0.0/0.0/2.0	**0.030**
Mesangial matrix expansion (mm)	82.8/12.1/2.0/3.0	86.1/5.0/1.0/7.9	0.058
Arteriolar hyalinosis (ah)	33.3/38.9/22.7/5.1	29.7/49.5/13.9/6.9	0.163
Interstitial fibrosis and tubular atrophy (IFTA)	3.10 ± 6.80	7.39 ± 13.09	**<0.001**
Thrombotic microangiopathy, %	5.6	14.9	**0.007**
Nephrocalcinosis (No/Mild Moderate/Severe), %	89.4/4.0/6.6/0.0	88.1/7.9/4.0/0.0	0.260
Tubular hypertrophy, %	18.7	27.7	0.073
Epithelial cell flattening (0/1/2/3), %	4.0/39.4/32.8/23.7	7.9/33.7/32.7/25.7	0.461
Brush border membrane defect (0/1/2/3), %	1.0/25.8/46.5/26.8	2.0/18.8/43.6/35.6	0.291
Vacuolization (0/1/2/3), %	7.6/22.2/21.2/49.0	4.0/24.8/20.8/50.5	0.660
Loss of nuclear staining (0/1/2/3), %	2.5/28.3/37.9/31.3	0.0/22.8/44.6/32.7	0.251
Cellular detritus (0/1/2/3), %	16.2/40.9/22.2/20.7	7.9/33.7/25.7/32.7	**0.036**

Continuous variables are presented as mean ± standard deviation. MW, mean value; SD, standard deviation.

Bold values represent statistically significant parameters.

The following categories of reasons for discard were recorded: 1) Macroscopic organ damage, such as renal capsule fissure, cortical hemorrhage, large infarcts, large renal cysts, heavy aortic patch and/or renal artery atherosclerosis and mottled appearance after reperfusion. 2) findings of procurement biopsies. 3) concerns about a transmissible donor infection, 4) extrarenal malignancy known or detected during procurement or tumor of the contralateral kidney; 5) denial of the transplant center to finally accept the offer 6) non transplantability of the recipient.

47 kidneys were discarded due to macroscopic findings, 43 due to the results of biopsy and 27 due to one of the reasons belonging to categories 3 to 6. Unfortunately, for nearly every fifth discarded kidney (32/149, 21.5%) the exact reason remained unknown.

### Score Performance in Transplanted Kidneys

The performance of the scores is shown in Table 8. Depending on missing data, up to 103 (46%) out of the 223 DDKs had to be excluded for the analysis of the endpoints.

**TABLE 8:** Previously published scores for the quality assessment of DDKs tested in this study including the endpoints they were designed for and their performance in the original publication.

Publication/Score	Endpoints	Performance in original publication	Performance in our cohort
Delayed graft function			
^15^ *Balaz et al*. _(n = 171)_	DGF	AUC (95% CI)_CIV Score_: 0.659 (0.606–0.710)	AUC (95% CI)_CIV Score_: 0.506 (0.417–0.595)
		AUC (95% CI)_CIV Score + donor age + cause of death_: 0.694 (0.642–0.743)	AUC (95% CI)_CIV Score + donor age + cause of death_: 0.490 (0.401–0.579)
^6^Chapal et al. _(n = 131)_	DGF	AUC (95% CI): 0.73 (0.68–0.77)	AUC (95% CI): 0.709 (0.617–0.801)
^a^ ^7^Irish et al. _(n = 223)_	DGF	AUC 0.704	AUC (95% CI): 0.684 (0.612–0.757)
^8^Jeldres et al. _(n = 223)_	DGF	AUC: 0.743	AUC (95% CI): 0.503 (0.423–0.582)
^11^Schold et al. _(n = 222)_	DGF	Rate of DGF	Rate of DGF
		Donor Grade I: 16.7%	Donor Grade I: 42.9%
		Donor Grade II: 23.1%	Donor Grade II: 70.0%
		Donor Grade III: 30.3%	Donor Grade III: 68.6%
		Donor Grade IV: 39.2%	Donor Grade IV: 61.2%
		Donor Grade V: 46.3%	Donor Grade V: 53.2%
		AUC (95% CI): NA	AUC (95% CI): 0.451 (0.373–0.530)
Graft survival			
^17^ *Navarro et al*. _(n = 223)_	5 years graft survival	HR (95% CI)_Full Score_: NA	HR (95% CI)_Full Score_: 1.501 (1.143–1.972)
		HR (95% CI)_Score >5 vs. ≤5_: 6.95 (1.57–30)	HR (95% CI)_Score >5 vs. ≤5_: 1.994 (0.975–4.079)
		AUC (95% CI)_Full Score_: NA	AUC (95% CI)_Full Score_: 0.617 (0.513–0.722)
		AUC (95% CI)_Score >5 vs. ≤5_: NA	AUC (95% CI)_Score >5 vs. ≤5_: 0.567 (0.462–0.673)
^19^Port et al. _(n = 223)_	1 and 3 years graft survival	1 year graft survival for RR < 1.7/≥1.7: 90.6/84.5%	1 year graft survival for RR <1.7/≥1.7: 91.0/80.7%
		AUC (95% CI)_1 year_: NA	AUC (95% CI)_1 year_: 0.662 (0.369–0.955)
		3 years graft survival for RR <1.7/≥1.7: 79.4/68.0%	3 years graft survival for RR <1.7/≥1.7: 87.5/75.8%
		AUC (95% CI)_3 years_: NA	AUC (95% CI)_3 years_: 0.603 (0.515–0.692)
^26^Rao et al. _(n = 223)_	1, 3, and 5 years graft survival	AUC (95% CI)_1 year_: NA	AUC (95% CI)_1 year_: 0.699 (0.459–0.939)
		AUC (95% CI)_3 years_: NA	AUC (95% CI)_3 years_: 0.557 (0.456–0.658)
		AUC (95% CI)_5 years_: NA	AUC (95% CI)_5 years_: 0.576 (0.474–0.679)
		5 years graft survival KDRI quintile 1: 82%	5 years graft survival KDRI quintile 1: 80.6%
		5 years graft survival for KDRI quintile 2: 79%	5 years graft survival for KDRI quintile 2: 73%
		5 years graft survival for KDRI quintile 3: NA	5 years graft survival for KDRI quintile 3: 79%
		5 years graft survival for KDRI quintile 4: NA	5 years graft survival for KDRI quintile 4: 76%
		5 years graft survival for KDRI quintile 5: 63%	5 years graft survival for KDRI quintile 5: 68%
^20^ *Snoeijs et al.*_(n = 171)_	5 years graft survival	AUC: 0.74	AUC (95% CI): 0.630 (0.513–0.746)
^16^ *Vusser et al.*_(n = 223)_	3 years graft survival	AUC (Historic cohort): 0.65	AUC (95% CI): 0.637 (0.538–0.736)
		AUC (Validation cohort): 0.70	
^16^ *Vusser et al.*_(n = 223)_	5 years graft survival	AUC (Historic cohort): 0.67	AUC (95% CI): 0.620 (0.524–0.717)
		AUC (Validation cohort): 0.81	
^19^ *Remuzzi et al*. _(n = 223)_	3 years graft survival	AUC: N/A	AUC (95% CI): 0.605 (0.501–0.709)
Graft function			
^14^ *Anglicheau et al.*_(n = 223)_	1 year graft function	AUC _eGFR < 25 mL/min at 1 year_: 0.84	AUC (95% CI) _eGFR < 25 mL/min at 1 year_: 0.649 (0.540–0.758)
^9^Nyberg et al. _(n = 223)_	1 year graft function	Mean creatinine clearance	Mean creatinine clearance
		Kidney Grade A: 61.1 mL/min	Kidney Grade A: 51.5 mL/min
		Kidney Grade B: 51.8 mL/min	Kidney Grade B: 42.7 mL/min
		Kidney Grade C: 42.6 mL/min	Kidney Grade C: 35.7 mL/min
		Kidney Grade D: 33.7 mL/min	Kidney Grade D: 34.8 mL/min
^18^ *Ortiz et al.*_(n = 171)_	1 and 2 years graft function	Kendall’s tau_1 year_: 0.277 (*p* = 0.0006)	Kendall’s tau_1 year_: 0.157 (*p* = 0.026)
		Kendall’s tau_2 years_: 0.286 (*p* = 0.0005)	Kendall’s tau_2 years_: NA
Patient survival			
^13^Foucher et al. _(n = 120)_	Patient Survival	AUC: 0.69	AUC (95% CI): 0.642 (0.548–0.736)

Abbreviations: aHR, adjusted hazard ratio; AUC, area under the receiver operating characteristic curve; CADI, chronic allograft damage index; CI, confidence interval; ECD, expanded criteria donor; eGFR, estimated glomerular filtration rate; HR, hazard ratio; KDRI, kidney donor risk index; NA, not available; RR, relative risk; SCR, standard criteria donor.

Pathological and combined clinical and pathological scores are in italics; the numbers correspond to the references in the revised manuscript.

^a^ The Irish score was applied without considering the parameter history of transition, which was not available in the majority of recipients.

Chapal and Irish had the best predictability for DGF with an AUC of 0.709 and 0.684, respectively, whereas Jeldres had an AUC of 0.503, Balaz of 0.506/0.490, and Schold of 0.451. For the prognostication of graft survival, the best-performing scores were of Rao and Port for 1 year with a significant AUC of 0.699 and 0.662, followed by de Vusser for 3 years, Snoeijs and de Vusser for 5 years with respective AUCs of 0.637, 0.630 and 0.620. Regarding graft function the trend was similar. Here, Navaro was acceptable, whereas the performance of Anglicheau poor (AUC 0.649) and the significance of Ortiz marginal (Kendall’s tau 1 year 0.157, *p* = 0.026). The predictive power of the EPTS score was poor (AUC 0.642).

### Score Performance in Discarded Kidneys

In another approach we tested the scores for the prediction of discards (Table 9). The best results for the comparison between bilateral discard and bilateral transplantation (column A vs. column C of Table 9) showed Balaz (1.80 vs. 1.11, *p* = 0.034), Snoeijs (4.55 vs. 3.12, *p* = 0.028), Remuzzi (*p* = 0.013) and Ortiz (4.36 vs. 2.83, *p* = 0.029). For the comparison between unilateral discard and bilateral transplantation (column B vs. column C of Table 9), Balaz <1 (*p* = 0.030), Navaro (*p* = 0.010) and Remuzzi (*p* = 0.011) came out to be significant.

**TABLE 9:** Performance of the investigated scores for the prediction of discards vs. transplantation.

Score	Both kidneys were discarded (n_kindeys_ = 104)	One kidney was transplanted, one kidney was discarded (n_kindeys_ = 90)	Both kidneys were transplanted (n_kindeys_ = 248)	Overall	*p*-value	*p*-value
				*p*-value	A vs. C	B vs. C
^15^ *CIV Score* (*Balaz et al.*)	1.80 ± 1.42	1.33 ± 1.07	1.11 ± 1.08	**<0.001**	**0.034**	0.233
^15^ *CIV Score* (*Balaz et al.*)*, (<1), %*	15.9	23.4	34.1	**0.012**	0.382	**0.030**
^15^ *Composite CIV Score* (*Balaz et al.*)*, %(0/1/2/3)*	4.3/31.9/49.3/14.5	9.4/25.0/46.9/18.8	4.2/30.5/52.7/12.6	0.583	0.533	0.804
^6^DGFS scoring system (Chapal et al.), Value	—	−0.1440 ± 0.7896	−0.1201 ± 0.7989	0.924	—	0.924
^6^DGFS scoring system (Chapal et al.), % (Low risk/medium risk/high risk)	—	36.4/54.5/9.1	33.3/61.7/5.0		—	0.808
^7^DGF risk calculator (Irish et al.), Points	—	210.6 ± 18.9	223.6 ± 28.6	0.092	—	0.092
^7^DGF risk calculator (Irish et al.), Probability of DGF (%)	—	19.9 ± 17.3	24.1 ± 20.0	0.303	—	0.303
^8^Jeldres scoring system (Jeldres et al.), Points	—	137.9 ± 31.2	131.3 ± 35.2	0.358	—	0.358
^8^Jeldres scoring system (Jeldres et al.), Probability of DGF (%)	—	48.5 ± 20.1	44.6 ± 21.6	0.370	—	0.370
^11^Schold Risk Index	—	1.05 ± 0.32	0.95 ± 0.35	0.190	—	0.190
^11^Schold Grade I-V	—	0.0/3.8/23.1/26.9/46.2	3.6/9.7/23.0/30.3/33.2		—	0.554
^17^ *Navarro Score* (≤*3/4-5/6-7/>7*)	59.6/13.5/10.6/16.3	62.2/22.2/6.7/8.9	69.8/15.3/10.1/4.8	**0.011**	0.165	**0.010**
^17^ *Navarro Score > 5, %*	26.9	15.6	14.9	**0.022**	0.080	0.947
^19^Port	1.96 ± 0.52	1.97 ± 0.51	1.96 ± 0.47	0.991	0.909	274
^26^Rao	—	1.33 ± 0.31	1.17 ± 0.43	0.054	—	0.106
^20^ *Snoeijs*	4.55 ± 3.47	3.36 ± 2.61	3.12 ± 2.39	**0.001**	**0.028**	0.387
^16^ *Vusser* (*3 years prediction*)	66.5 ± 16.8	64.0 ± 17.4	62.6 ± 17.7	0.158	0.315	0.185
^16^ *Vusser* (*5 years prediction*)	63.2 ± 14.8	62.3 ± 16.0	61.2 ± 16.7	0.581	0.693	0.240
^19^ *Remuzzi Score* (*pirani*)	2.41 ± 2.76	1.90 ± 1.94	1.55 ± 1.84	**0.002**	0.141	**0.011**
^19^ *Remuzzi Grading* (*Score 1-3/4-6/7-12*) *(pirani) 1-3: for single transplantation, 4-6: for dual transplantation*	67.3/24.0/8.7	83.3/15.6/1.1	85.1/12.9/2.0	**0.001**	**0.013**	0.715
^14^ *Anglicheau* (*GS−/CP−; GS−/CP+; GS+/CP−; GS+/CP+*)	29.8/46.2/2.9/21.2	16.7/64.4/1.1/17.8	20.6/52.8/6.0/20.6	0.060	0.058	0.500
^9^Nyberg Score	—	26.1 ± 7.1	24.1 ± 9.0	0.261	—	0.261
^9^Nyberg Grading (A/B/C/D)	—	0.0/25.0/25.0/50.0	9.1/23.4/28.9/38.6			0.318
^18^ *Ortiz*	4.36 ± 2.91	3.34 ± 2.35	2.83 ± 1.98	**<0.001**	**0.029**	0.148
^13^Foucher	—	9.56 ± 2.90	8.39 ± 2.04	0.236	—	0.240

CIV, chronic interstitial and vascular score according to the Banff classification; composite CIV Score: CIV score considering also clinical parameters (donor age >51 years, anoxic donor brain injury).

A, B and C refer to the first (bilateral discard), second (unilateral discard) and third (bilateral transplantation) column of the table.

Pathological and combined clinical and pathological scores are in italics, the numbers correspond to the references of the manuscript.

Bold values represent statistically significant parameters.

## Discussion

Primary aim of this retrospective study was to test the performance of scores previously devised for quality assessment of a DDK of lower quality for their value in supporting the decision about discard or acceptance. The rather dismal clinical outcome in our cohort with 48.9% and 15.8% of recipients respectively developing DGF or losing their graft within the first year shows that it was indeed a formidable real-life challenge for the scores.

For DGF we found an acceptable discrimination with an AUC of 0.709 for the Chapal score. The Irish score could have even performed better if we would have been able to provide the missing recipient parameter of “previous blood transfusion.” Moreover, the applicability of the purely clinical and thus economical Irish score is limited because it requires the cold and warm ischemia time, both unknow at the time of allocation. Conversely, the Chapal score required donor- and recipient parameters, which, except for the cold ischemia time, are easily to obtain. The score of Chapal showed a lower AUC than that reported in the initial publication [6]. This may be explained by the higher incidence of DGF in our cohort (48.9% vs. 25.4% reported by Chapal).

Similarly poor results were seen for the Anglicheau and Ortiz scores to predict graft function. Their poor performance may be explained by the higher age of our recipients, compared with those in the cohorts of Anglicheau and Ortiz (61.0 vs. 50.6 vs. 48 years), as well as the higher ratio of our donors with hypertension (56.8% vs. 30.8%) and their higher creatinine levels before organ removal (149 vs. 101 μmol/L) compared with those in the cohort of Anglicheau. However, the better performing score of Nyberg, requires cold ischemia time, a parameter not known at the time of allocation.

None of the scores for graft survival reached an acceptable performance. The pathological scores of Navarro and Snoejjs and the clinicopathological of de Vusser outperformed the solely clinical Rao and Port’s scoring systems. This suggests that there are aspects of donor organ quality that cannot be reliably determined from clinical data alone. Inclusion of pathologic data could allow for better assessment of overall organ quality, particularly in kidneys of lower-than-average quality and explain the better performance of the scores with histopathology. Still, this was not sufficient to push AUC into the acceptable range. The score of Navarro [17] has been adopted by the Spanish Society of Nephrology [46]. Here, kidneys with a score <8 are proposed for single transplantation. The very poor results obtained by Navarro et al in their study transplanting kidneys with a score 6–7 were not confirmed later by others [47].

In summary, the majority of the scores are not suitable for procurement biopsies because they include information, which is not available during procurement. Beyond that, the scores were developed after examination of paraffin embedded renal tissue, a procedure that is time consuming and not practical in the limited time setting of allocation. The only exception is the Remuzzi score, which was based on frozen sections. However, in our experience frozen sections are often difficult to evaluate due to inappropriate handling during transport [31].

Procurement may also lead to needless discards if the histopathologic evaluation is conducted by general pathologists and not by nephropathologists. The failure of pretransplant biopsies to predict graft outcomes was highlighted in an older metaanalysis of 47 studies testing 15 scores [48]. In a recent paper, more than half of kidneys discarded in US would have been suitable for transplant in France, where procurement biopsies are rarely performed [49]. Furthermore, their usefulness has been questioned due to low reproducibility and poor predictive power [50], albeit there are centers proposing punch- instead of wedge or needle- biopsies as a means to improve standardization, sample adequacy and reproducibility [51]. At all, scores based on preimplantation biopsies can be implemented to predict graft function but their applicability to decide on transplantation or discard has probably been overestimated [52].

Strengths of our study were the comprehensive evaluation exclusively of procurement biopsies by an experienced nephropathologist according to the most recent Banff criteria [29] and the validation of the most known scores for the endpoints for which they have been developed.

Limitations should also be recognized. **First**, the definition of DGF as need for dialysis within the first week after transplantation, an endpoint that may be influenced by various clinical factors (such as heart failure, hyperkalemia, etc.) is not uniformly accepted. Furthermore, we excluded PNF, because it has a different pathogenesis [40] and was not tested as outcome parameter in the scores. The extraordinarily high incidence of PNF and DGF was probably due to bias by indication; our cohort was highly selective since biopsies were performed only in those donors whose organs were supposed to be of lower quality. Another reason was the higher incidence of donors with AKI an acknowledged risk factor for both outcomes [53]. **Second**, the scores have been constructed on preimplantation biopsies, which are in terms of prognostication completely different from procurement biopsies due to the accrued damage during cold preservation and transport as well as the reperfusion injury after implantation. **Third,** the number of missing data implies that each score was tested on different or partially overlapping sub-cohorts. However, this problem is unavoidable, since the data required for the calculation of all scores, are not routinely collected in the ET database nor at the DSO or the transplant centers. A registry with data of all sources (DSO, ET, transplant centers) is not available. **Fourth**, the test cohort dates back approximately 10 years. However, most of the evidence base of kidney transplantation relies on data collected before 2010 and the follow-up period of our study should not have changed considerably in the decade before and after 2010.^4^ **Fifth**, the indications for procurement biopsies relied not on objective criteria since they were performed on case-by- case basis and not according to a standardized protocol. For example, the macroscopic assessment of the recovered organs was quite subjective. However, it can be of value if performed in a more structured way by experienced surgeons [54]. **Finally**, an inherent, unavoidable drawback of all similar studies is the unknown performance of the certainly non-randomly discarded DDKs. Despite all these limitations, this is the only study examining the performance of these scores on the dataset for which they are most usefully from a clinical point of view: procurement biopsies for the decision of DDK transplantation or discard. We found that, that none of the tested scores should allow a confident, evidence-based decision about acceptance or discard of a DDK based on prognosis of the different endpoints within the ET context. Probably, clinical parameters not included in that scores, such as donor’s AKI or donor’s creatinine metrics are more important for short term outcomes [53, 55].

Here, some conclusions can be drawn: **First**, organs from donors with AKI should not be accepted for recipients at high risk for DGF or these recipients may be preferentially treated with an immunosuppression protocol based on belatacept [56]. **Second**, the recipient should return timely to dialysis to avoid losing it waitlist points if an early graft failure is expected. **Finally**, we must always keep in mind that especially for the elderly patients, rejection of organs leads in the end to an increase in mortality due to the longer waiting list time [57].

Regarding the second aim, we could indeed show that for the endpoint death censored graft survival histological [17, 20], or clinicopathological [16] scores performed marginally better than purely clinical ones. But even if the AUCs were slightly better their overall performance was moderate to poor. While for some DDKs donor and recipient parameters might be entirely sufficient for a prognosis, for some donor/recipient matches histopathology might add valuable information*.* We are currently investigating such an approach with a facultative histopathology component including only reproducible parameters independently from each other associated with prognosis.

As to the testing of the scores in the discarded kidneys, we found that scores with a histological component were better than the solely clinical. However, an inherent bias cannot be excluded since the histologic evaluation of an offered organ is often the principal reason of its discard. Here, we can only postulate that histological assessment is warranted in kidneys supposed to be unsuitable for transplantation. Probably, the most important finding was that many of the discarded kidneys could have been successfully transplanted.

## Conclusion

Procurement biopsies are often used during allocation to increase the possibility of acceptance of kidneys of lower quality. However, the available prognostic scores perform at best only moderately. Though none of the scores could reach an acceptable discriminatory power, those based on histopathologic criteria performed slightly better than the more practical solely clinical ones. Our findings are based on data from the Eurotransplant region but can also be applied to other Multinational or National Transplant Organizations or -even more- be valuable for individual decisions in transplant centers.

## Data Availability

The raw data supporting the conclusion of this article will be made available by the authors, without undue reservation.

## Footnotes

1
www.Eurotransplant.org

2
https://optn.transplant.hrsa.gov/media/1512/guide_to_calculating_interpreting_kdpi.pdf

3
hrsa.gov

4
https://www.ctstransplant.org/public/introduction.shtml
