# Supplementary material for: Performance of Scores Predicting Adverse Outcomes in Procurement Kidney Biopsies From Deceased Donors With Organs of Lower-Than-Average Quality
Source: Transpl Int. 2023 Oct 12;36:11399. doi: 10.3389/ti.2023.11399 (PMC10600346; doi:10.3389/ti.2023.11399)
Supplement: Supplementary file 1 [file Table1.docx]

**Supplementary file:** Parameters considered in the histopathologic evaluation of procurement biopsies (Table was created by using data from the references 29 and 31-37 of the revised manuscript)

| **Parameter** |
| --- |
| **Glomerular compartment** |
| Total Number of glomeruli |
| Number of globally sclerosed glomeruli |
| Number of segmentally sclerosed glomeruli |
| Type of segmental glomerulosclerosis; NOS, perihilar, collapsing, cellular, tip lesion |
| Mesangioproliferation: no/focal/diffuse) |
| Banff components ***g, mm, cg***: g0,g1-g3, mm0, mm1-3, cg0,cg1-3 |
| DN according to RPS (I,IIA, IIB, III or IV) |
| **Arteries and arterioles** |
| Number of arteries |
| Severity of intimal fibrosis (cv): cv0, cv1-3 |
| Severity of hyalinosis (***ah***), ah0, ah1-3 |
| Fibrosis of small preglomerular arteries(***fib***): fib0, fib1-3 |
| Arteriitis: v0, v1-3 |
| Glomerular and /or preglomerular Microthrombi: focal/diffuse |
| Peritubular capillaritis: ptc0 ptc1-3 |
| **Tubulointerstitial compartment** |
| Banff components ***ci*** and ***ct:*** ci0, ci1-3, ct0, ct1-3 |
| Banff components ***t***, ***i***: t=0. t1-3, i=0, i1-3 |
| IFTA score: 0 (<10%), 1(10-25%), 2 (26-50%), 3 (>50%) |
| Nephrocalcinosis: present/absent |
